# Effect of Polymeric Moisture Barriers on ZnO Nanofiber Gas Sensors Operating at Room Temperature

**DOI:** 10.1021/acsomega.5c00664

**Published:** 2025-04-08

**Authors:** Selda Topcu Sendogdular, Levent Sendogdular

**Affiliations:** Department of Metallurgical and Material Engineering, Erciyes University, Kayseri 38039, Türkiye

## Abstract

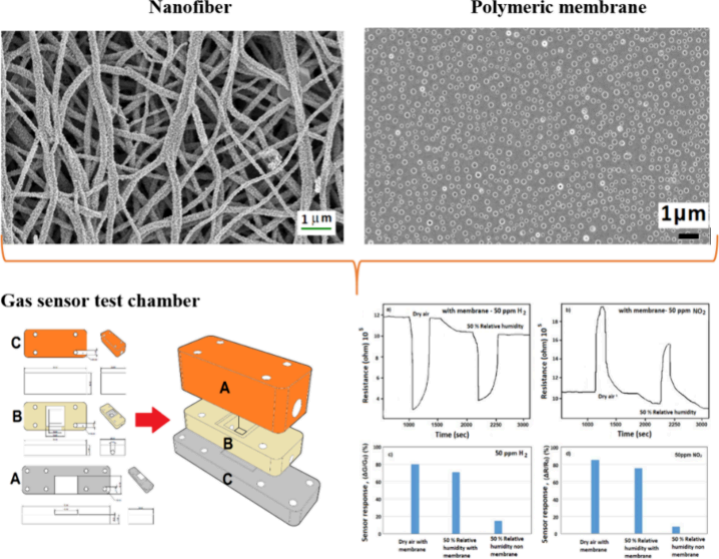

Relative humidity is a significant factor that impairs gas sensor performance. Some investigations have heated sensors to temperatures beyond 100 °C to eliminate relative humidity, negatively affecting the sensor stability and application range. To improve the sensitivity of gas sensors that operate at room temperatures, free-standing polymer films with moisture barrier qualities are applied to various sensor architectures. A selective permeable polymer membrane applied to the air interface of the sensors, with or without contact, is intended to lessen the effects of relative humidity on sensor sensitivity. In this study, ZnO structures were synthesized by using electrospinning methods. Selective permeable polystyrene/poly(ethylene glycol) (PS/PEG) polymer film membranes were produced on the synthesized nanofiber structures and applied to the sensor. In the membrane synthesis, the CO_2_ annealing process was applied to control the porosity O_2_ and moisture permeability. Gas sensor performance tests for NO_2_ and H_2_ gases were conducted for these synthesized nanostructures and membranes by using various characterization techniques and analyses. Gas-sensing measurements were performed in dry air and a relative humidity (RH) of 50%, employing different concentrations of NO_2_ and H_2_ gases. Different sensing parameters (response time, recovery time, sensitivity) were estimated at room temperature for samples, and the sensor sensitivity was 0.0152 at 50 ppm. Sensor response is enhanced approximately fivefold by samples with polymeric membrane measurements compared to without. Nanofibers exhibit 120 and 300 s response time and recovery time for NO_2_ gas, respectively. As a result, a new approach to the literature has been provided to reduce the effects of RH on the sensor, which is one of the biggest obstacles in the scope of gas sensors operating at room temperature. Therefore, this study’s findings open a general approach for fabricating flexible devices for gas detection applications.

## 1 Introduction

Environmental problems have emerged with increasing consumption due to the growing population, technological developments, and social welfare. Today, gas sensors are used in many areas ranging from carbon emission control to energy production. Gas sensors operating at room temperature have attracted attention with their increased reliability due to their stable structure, as well as their low energy consumption and simple manufacturing.^1,2^ The lack of heating elements, considered the most essential feature of new-generation gas sensors, directly affects their cost, reliability, and applicability.^3^ Gas sensors operating at room temperature also find a place in strategic areas such as the measurement and control of automobile and factory emissions that pose a danger to human health and flammable and explosive gases that are undesirable in working environments,^4^ food freshness control,^5^ and early medical diagnosis from human breath.^6^

Alternative semiconductor materials and structural arrangements are essential in the development of gas sensors. Semiconductor metal oxide gas sensors are among the priority areas due to their high gas response, excellent selectivity, good portability, and low production cost.^7^ With the development of nanotechnology, nanoscale semiconductor materials can be obtained so that technologies with higher sensitivity^7^ can be produced, and sensors’ applicability and response speed can be improved due to their small size.

ZnO, a well-known n-type semiconductor among metal oxides, is known to be an excellent gas-sensing material for the detection of both reducing^8,9^ and oxidizing gases.^10^ They possess the properties required for an ideal gas sensor, such as a wide band gap (3.37 eV), high electron mobility (210 cm^2^/(V s)), and excellent chemical and thermal stability.^11−13^ The gas sensing of ZnO is determined by the change in sensor resistance caused by the electrochemical reaction of gas molecules on the sensor surface.^14^ Therefore, ZnO nanofiber structures have been the subject of this study as they improve the gas-sensing properties due to their large surface-to-volume ratio and highly reactive sites, enhancing the sensing capability over high gas adsorption. The most significant disadvantage of gas sensors operating at room temperature is the RH of the environment. RH decreases the sensitivity of the gas sensors. Different approaches such as surface modification,^15,16^ doping,^17−19^ preparation of hybrid/composite nanomaterials,^20^ self-heating effect,^21^ and light activation^22,23^ are available in the literature to reduce the operational temperature in gas sensors; however, these studies are disadvantageous in terms of temperature, sensor stability, and scope of application.^24,25^

Usually, oxygen is adsorbed close to the oxygen vacancy, which is essential for ZnO’s sensing mechanism. Water molecules are also adsorbed on the oxygen molecules; therefore, the adsorbed oxygen and water molecules reach a dynamic equilibrium. As the humidity increases, the equilibrium condition is broken, more water molecules are adsorbed, and the density of adsorbed oxygen decreases, thus reducing sensor response.^1^ At this stage, RH is the most crucial critical threshold to overcome.^26,27^ Therefore, in this study, a selectively permeable polymer moisture barrier was applied to the sensor to compensate for the effect of humidity in sensors operating at room temperature.

Polymeric materials are used in various applications, from packaging materials as moisture barriers (such as food, pharmaceuticals, and microelectronic systems) to moisture-resistant materials, corrosion barrier films, and reverse osmosis membranes. Polymer packaging is a key factor in determining shelf life, especially in the food sector.^28^

It is known that barrier performance can be determined by the material’s structure and the barrier’s molecular porosity.^29,30^ While improving the moisture barrier property is desirable within the study’s parameters, suppressing the oxygen barrier property is also sought to enhance the number of oxygen and analyte gas molecules that can be adsorbed on the sensor surface. In other words, even though the membrane has a high oxygen permeability, it should have a low moisture permeability. On the other hand, materials with substantial oxygen barriers generally have polar–polar interactions or hydrogen-bonded molecular structures, which cause the structure to have high hydrophilicity. Conversely, materials with poor oxygen barrier properties provide successful results, such as water vapor barriers.^31,32^ In this study, polystyrene (PS) with moisture permeability between 400 and 1000 g μm/m^2^ day kPa and oxygen permeability between 100,000 and 150,000 cm^3^ μm/m^2^ gün atm was preferred as a good barrier material as a nonpolar polymer.^33−36^ Hence, the structural density (porosity) determines gas permeability. PS/PEG systems were utilized to control porosity, which is achieved by phase separation of a blend of two different polymers insoluble in each other.^37^

For the free polymer membrane film, the CO_2_ annealing method based on water repellency^38^ and/or physical nano/microporosity^39,40^ was used. CO_2_ annealing is also a practical postprocessing method to tune the surface energy of polymers. It is known that both CO_2_-soluble and CO_2_-insoluble polymer chains expand under supercritical CO_2_ in dilute systems.^41^ The increase in these polymer chain correlation lengths in the density fluctuation region is explained by the increase in the entropic component of the ground-state equation. For the CO_2_-insoluble polymers, this increase is thought to cover part of the unsupported mixing entropy, leading to anomalous chain expansion.^42−47^ These anomalous expansions were found to be directly related to fluctuations in the density of supercritical CO_2_.^48,49,43,50^ It has been proven that the macro properties of the polymer thin film and even polymer bulk structures can be modified through this expansion.^51−54^

The scope of the study is based on the synthesis of free polymer films with moisture barrier properties to increase the sensitivity of gas sensors operating at room temperature and the application of these membranes on ZnO nanofiber sensor structures synthesized by the electrospinning method. Gas sensor performance tests were carried out for NO_2_ and H_2_ gases with selectively permeable PS/PEG membranes subjected to CO_2_ annealing through a system designed specifically for this study. Within the project’s scope, the aim is to reduce the effects of RH on sensor sensitivity with the selectively permeable polymer membrane applied to the air interface of the sensors.

## 2 Experimental Methods

### 2.1 Materials

Polyacrylonitrile (PAN, Mw ∼ 150,000 g/mol), DMF (Mw ∼ 73.09 g/mol), polyvinylpyrrolidone (PVP, Mw ∼ 1,300,000 g/mol), zinc acetate (ZnAc, Mw ∼183.47 g/mol), PS (Mw ∼192,000 g/mol), and polyethylene glycol (PEG Mw ∼35,000 g/mol) chemicals were obtained from Sigma-Aldrich.

### 2.2 Preparation of Nanofiber

2.23 g of PVA (2.23 g) was first dissolved in 12 mL of DMF to synthesize nanofibers. After dissolving 4.5 g of PVP and 1.2 g of ZnAc (Zinc acetate) in 25 mL of DMF, these two solutions were mixed in a beaker with a magnetic stirrer for half an hour until a homogeneous sol–gel form was achieved for electrospinning. The sol–gel solution was placed in a 10 mL syringe, and the syringe was fixed to a feeder pump set at a speed of 1 μl/min. A conductive metal needle (22 gauge) was attached to the tip of the syringe. The positive electrode of the power supply was connected to the metal needle tip, and the negative and ground electrodes were connected to the collector aluminum foil. The voltage applied from the power supply was set to 20 kV, and the distance between the needle tip and the collector was set to 20 cm. The ambient temperature was kept constant at room temperature. After continuous and homogeneous fibers were synthesized, they were placed in a furnace to remove organic contents, such as polymeric binders, over the calcination temperature. The heating rate was 25 °C/h, and samples were kept at peak temperature (650 °C) for 2 h. Calcination is also necessary to increase the surface area of zinc oxide nanofibers by removing the matrix phase (polymeric binder).

### 2.3 Synthesis of Polymeric Membrane

PEG/PS solutions with a 1:10 weight ratio were prepared for membrane synthesis. Due to the low boiling point of toluene, the solution was heated in a magnetic stirrer at 50 °C for about 2 h until a transparent solution was obtained. The solution, whose homogeneity was visibly determined, was then a thin film synthesized by spin coating at 3000 rpm for 1 min and 1000 rpm for 30 s. The spin-coated films were then separated from the substrate using a floating technique to synthesize free-standing thin films.^37^ The films were kept in temperature-controlled (∼30 °C) pure water for 10–15 min to remove the PEG chains. The films were then transferred onto a glass substrate and dried with nitrogen. In addition, the CO_2_ annealing process was applied to thin films on a glass substrate. The CO_2_ annealing method has been described in more detail in previous studies.^55−57^ CO_2_ annealing was applied in a high-pressure cell at 36 °C and 8 MPa for 24 h.

### 2.4 Characterization Techniques

Optical microscopy (ZEISS Axio Lab A1), atomic force microscopy (AFM, Veeco Multimode 8), and scanning electron microscopy (SEM, Gemini 500, Zeiss) measurements were applied to investigate the surface morphology and topography. As preparation before SEM, the dried samples were coated with a target consisting of 80% gold (Au) and 20% palladium (Pd) by sputtering method at a rate of 3Å/s for 15 s to obtain a thickness of 45Å.

The structure of nanofibers was examined with an X-ray diffractometer (XRD Bruker AXSD8 Advance Model) equipped with Cu–Kα radiation source (λ = 0.154 nm), and the scanning range (2θ) was set between 10° and 90° for 0.002 °C/s scan rate. AFM measurements were captured with a VEECO multimode 8 device. Scanning was performed in tapping mode with a speed of 1 Hz. An area of 40 × 40 μm was scanned. UV–vis spectroscopy (UV-1800, Shimadzu) was used to measure the absorbance of additives in the nanofibers. Diffuse reflection spectroscopy was converted into absorption mode *F*(*R*) using the following Kubelka–Munk function equation,^58,59^ where *R* is the absolute reflectance of the samples and is defined as the Kubelka–Munk function:
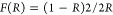
1

The Tauc plot was applied according to the following equation to estimate the band gap energy of the synthesized ZnO nanostructures:
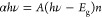
2where *A* is a constant; *n* is an index equal to 2 and 1/2 for indirect and direct allowed transitions, respectively; and *E*_g_ is the band gap energy of the material. The versus curve, the Tauc plot, is obtained using eq 2, which is necessary to calculate the materials’ band gap energy.^60^ Band gap energy is also equivalent to the absorption coefficient (α); however, a more precise analysis can be reached by extrapolation of the linear part of the Tauc plot.

Contact angle measurements were performed by using an optical tensiometer (Attension Theta Lite) to measure the hydrophobicity of the membranes.

### 2.5 Oxygen and Moisture Permeability Test

The test followed the ASTM D3985 standard in the Labthink TOY-C2 brand oxygen permeability device. Nitrogen was preferred as carrier gas (10 mL/min N2) at 20–25 °C and 20–25% RH. The measurement was not initiated until the oxygen transmission rate stabilized. When the measurement was completed, the oxygen transmission rate was multiplied by the film thickness and the oxygen permeability value was calculated in ml cm/m^2^ day units.

The moisture permeability test applied to thin membrane materials was carried out under the ASTM F1249 standard test method^61^ using the Labthink TSY-T3 device. The calibrated device works with the weight method. Therefore, the balance was calibrated, and the replacement silica gels were tested with the calibration sample to confirm the accuracy of the measurements. The measurements were carried out under the recommended temperature of 38 °C and an RH of approximately 90%. The moisture permeation rate obtained was again multiplied by the film thickness (500–1000 nm) to determine the moisture permeation value (g cm/m^2^ day).

### 2.6 Device Fabrication and Sensor Testing

**Figure 1:**
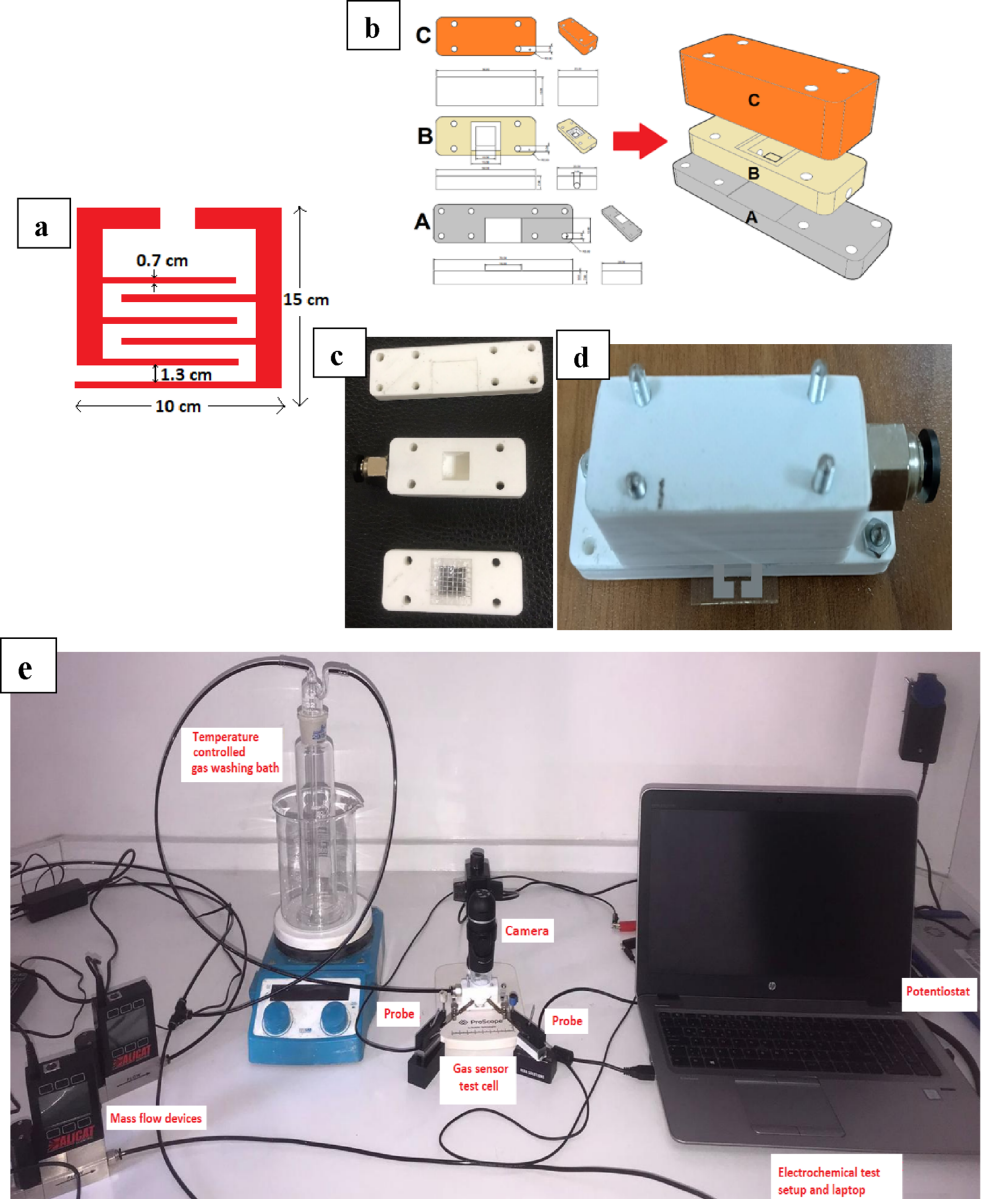
(a) Schematic dimensions of the contact and (b) design of the test chamber: A sensor holder, B membrane holder, C gas supply; (c) sequence of parts of the test cell, (d) test cell, and (e) gas-sensing experimental setup.

A Kapton tape was placed onto the glass substrate as a mask material before coating the platinum using the PVD technique. The Flux brand laser cutter (Figure 1a) was used to engrave the shape of contacts on the Kapton tape stuck to the glass substrate. Then, the processed glass substrates were coated with platinum in a PVD device at a speed of 0.4 Å/s, 90 W power, with a thickness of 200 nm. The Kapton tape was removed, leaving the desired platinum contact form on the glass substrate. After being coated, the synthesized nanofiber was stabilized on the glass substrate. In this context, 0.1 g of ZnO sample was dispersed in 10 μL of deionized ethanol. The solution was applied in drops onto an ITO glass electrode and kept at 50 °C for 2 h. The thickness of the sensing layer was measured to be approximately 30 μm.^62^

The Ivium Compactstat model potentiostat was used for gas sensor test measurements (Figure 1e). Measurements were conducted in dry and humid air (50% RH). A gas-washing bath was used for humid air measurements. The amount of water the air can carry depending on the temperature was taken as a basis to control the RH of the air passed through the gas-washing bath. CAD drawing images of the sensor test cell are shown in Figure 1b,c,d. Within this design’s scope, the platinum electrodes’ feet were left outside to ensure contact with the needle-type tungsten probes of the potentiostat. This setup was specially designed for this project, and the volume left inside the test cell was fixed at 1 cm^3^. This simplified calculating and controlling parameters such as gas flow rate and contact surface area.

Polymeric membranes were mounted to avoid direct touch with the sensor device using a wire mesh to prevent possible blockage of the sensor’s active surface area. During assembly, the membrane placed on the wire mesh substrate was fixed to the test cell. Gas sensor tests were carried out with the two-contact potentiostat measurement technique. In this context, tungsten needle contacts with a diameter of 25 μm and an approach angle of 45 ° supplied by Rera Solutions were used for sensitive conductivity measurements of semiconductors.

The gas sensor’s performance was evaluated based on resistance and gas measurement responses. The sensor tests were performed at room temperature under a reductive analyte gas (H_2_) and an oxidizing analyte gas (NO_2_). The response of each sensor was obtained from resistance versus time plots. It is defined as the response of the sensor:
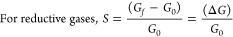
3where *G*_0_ is the conductivity of the semiconductor in an atmospheric environment, and *G_f_* is the conductivity value measured under gas flow.
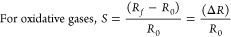
4where *R*_0_ is the resistance value measured in the atmospheric environment, and *R_f_* is the resistance value measured under gas flow.

The time it takes to accomplish 90% of the total resistance change in the case of adsorption and desorption, respectively, was used to define the response and recovery times for the sensor.

## 3 Results and Discussion

**Figure 2:**
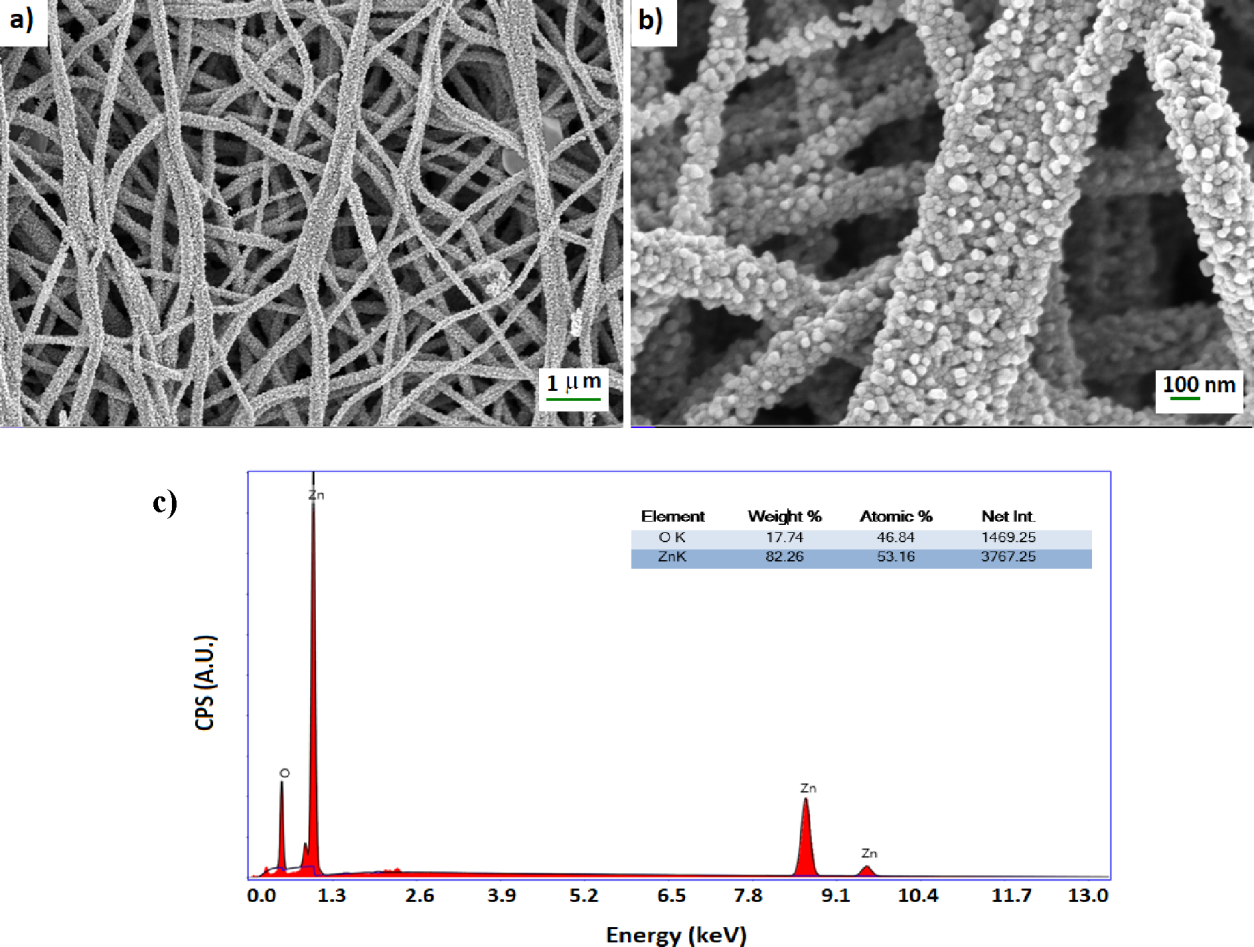
(a,b) SEM images at different magnifications and (c) EDX analysis of the calcined nanofibers synthesized by electrospinning.

SEM images of the prepared nanofibers at different magnifications after calcination are also given in Figure 2.

The diameters of ZnO fibers range from 200 to 300 nm. Each nanofiber comprises tiny grains, or nanograins, about 25 nm in diameter, as seen in Figure 2a,b, a high magnification image. In addition to grain growth, higher annealing temperatures have the effect of uniform distribution of grains uniformly. The size of the nanograins probably determines all of the physical and chemical characteristics of the nanofibers. It was also known that the nanofibers’ high-temperature calcination revealed the nanograins’ growth.^63^

The nanofibers were made of ZnO material since the energy-dispersive X-ray (EDX) spectroscopy composition analysis in Figure 2c revealed that only the peaks associated with Zn and O atoms (the molar ratio of Zn to O was almost 1) were observed.

**Figure 3:**
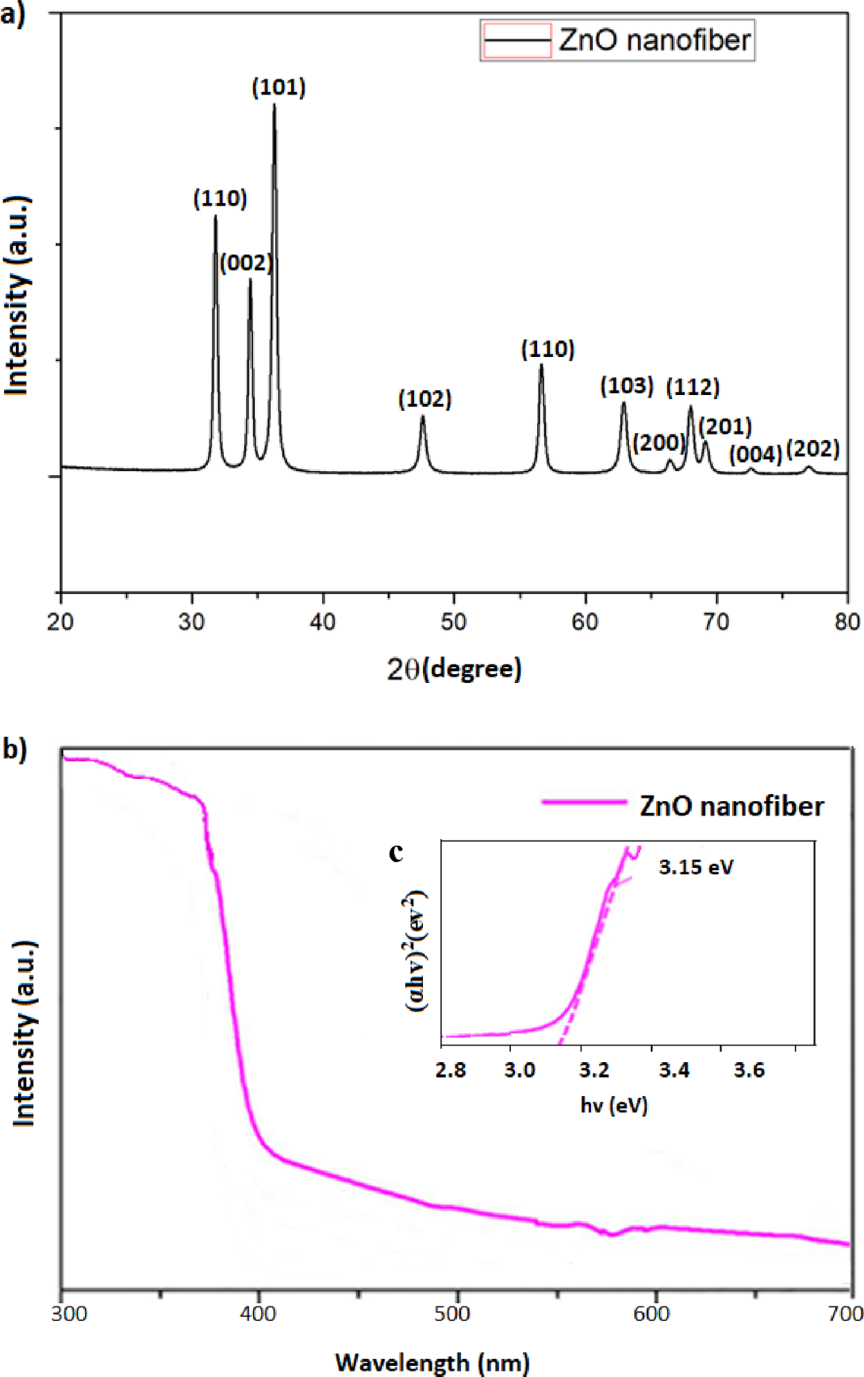
(a) XRD analysis of the nanofiber synthesized by electrospinning. (b) UV–vis spectrophotometer plot and (c) TAUC plot diagram of the ZnO nanofiber.

Figure 3 shows XRD images of the synthesized nanofibers. The results show that diffraction peaks appear at 31.7°, 34.3°, 36.1°, 47.5°, 56.6°, 63.1°, 66.4°, 67.8° 69.0°, 72.5°, and 77.1° corresponding to (100), (002), (101), (102), (110), (103), (200), (112), (201), (004), and (202) planes. When the peaks in these graphs were analyzed, it was understood that the ZnO structure following PDF no. 36-1451 was obtained. ZnO nanofibers annealed at 650 °C show diffraction peaks originating from (110), (002), (101) and (102) planes, which characterizes the wurtzite ZnO. No other impurity-related diffraction peaks were found. Therefore, it was evident from the data that the product was pure ZnO nanofibers.

To better understand the structure-dependent band gap energy of the semiconductor ZnO nanofiber and thus the crystal arrangement, tests were carried out in a UV–vis spectrophotometer device. After determining the light absorption of the samples in the wavelength range of 200–600 nm, the band gap energy (*E*_g_) was calculated. The relevant UV–vis spectrophotometer graph of the nanofiber samples is shown in Figure 3b. In Figure 3c, the band gap energies calculated by the Tauc graph^64^ were determined. According to the results, the optical band gap energy of the nanofibers is 3.15 eV.

SEM images show that the pores are homogeneously distributed; while the average size of the pores before CO_2_ is around 200 nm, the average pore size declines to 100 nm.

Apart from fluorinated polymers known to dissolve under CO_2_, CO_2_ is known to be a suitable solvent, especially for thermoplastic polymers, and polymers are known to expand by extending their chain lengths over the increasing radius of gyration (Rg) in a suitable solvent due to chain conformations.^43^ Polymer/solvent interaction is reportedly most substantial in the supercritical region, where fluctuations in CO_2_ concentration occur. Changes in the chain structure are known to reduce the density of polymers while bringing macro properties such as water repellency and self-repair.^65,66^ Previous studies in the literature for PS and PEO thin films have shown that maximum polymer expansion (∼15%) is achieved in the region corresponding to 8 MPa CO_2_ pressure.^46^ In addition, water contact angle studies have also shown that entropic changes regarding the chain conformations can tune the water repellency of polymers annealed in this region.^11^

Under supercritical CO_2_, polymer chain entropy increases, since the fluid is a suitable solvent, which leads to the elongation of the length of the chains and the strengthening of the interface. Water repellency also rises as a result of the reducing surface energy of the polymer chains, which increases mobility after CO_2_ and reaches the equilibrium state since supercritical CO_2_ annealing is a plasticizing process. The supercritical conditions of CO_2_ described by Li et al. were established as parameters by considering the chain entropy and the resulting water repellency and polymer density that change with chain elongation.^46^ Accordingly, it is thought that the 24 h equilibrium period determined in previous studies will provide optimum conditions.^43^

**Figure 4:**
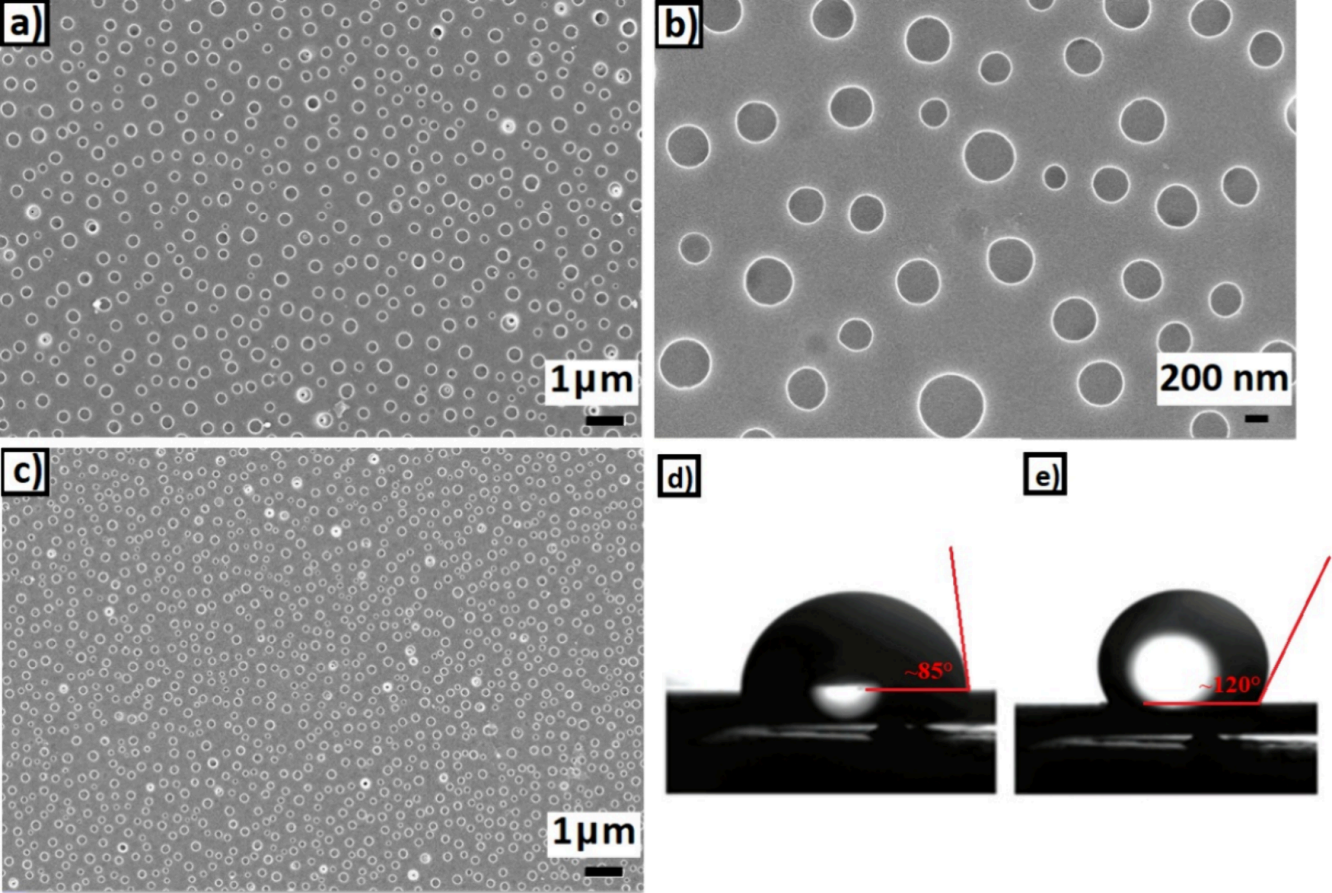
(a,b) SEM images of 500 nm PS/PEG (10:1) films before and (c) after CO_2_ annealing. Water contact angle images of the films (d) before and (e) after 36 °C/8 MPa CO_2_ annealing.

Figure 4 illustrates the SEM images of 500 nm PS/PEG (10:1) films before CO_2_ annealing with different magnifications (Figure 4a,b) and after CO_2_ annealing (Figure 4c). According to the SEM images, it is seen that the pore diameter decreased by nearly 50%. Thickness measurements performed with an ellipsometer also show that the film thickness, which was 500 nm before CO_2_ (Figure 4a,b), was swollen up to 580 nm average after CO_2_ (Figure4c). This is in direct proportion to the expansion measurements given in the literature. The connection between the 15 and 16% expansion obtained vertically and the nearly 50% expansion observed through the pores is thought to be related to the CO_2_ diffusion depth.

The diffusion depth of supercritical CO_2_ is around 100–150 nm, according to previous neutron synchrotron studies.^11^ Consequently, the response of roughly 100 nm of the film is the expansion acquired vertically, that is, in the direction of CO_2_ diffusion. However, the polymer/CO_2_ interaction is stronger around pores because the air interface is broader. Consequently, Figure 4 shows that in the porous sections, CO_2_ at the supercritical state creates an interface through the 500 nm film thickness in the region corresponding to the pore walls. This depth is equivalent to fivefold of the supercritical CO_2_ diffusion depth. As a result, it is understood that the contribution of the polymer chains around pores to the anomalous dilation is far more significant than the ones on the surface.^57^

The water contact angle measurements of the synthesized membrane before and after the addition of CO_2_ are displayed in Figure 4d,e. Contact angles less than 90° are considered hydrophilic, between 90 and 150° are considered hydrophobic, and contact angles greater than 150° are considered superhydrophobic.^67^ Based on the pertinent results, the water contact angle, or hydrophobicity, rose under a CO_2_ pressure. The water repellency of films annealed under supercritical CO_2_ approaches the optimal level after 24 h. These findings indicate that after CO_2_, the membranes’ water repellency rose by about 40%.

**Figure 5:**
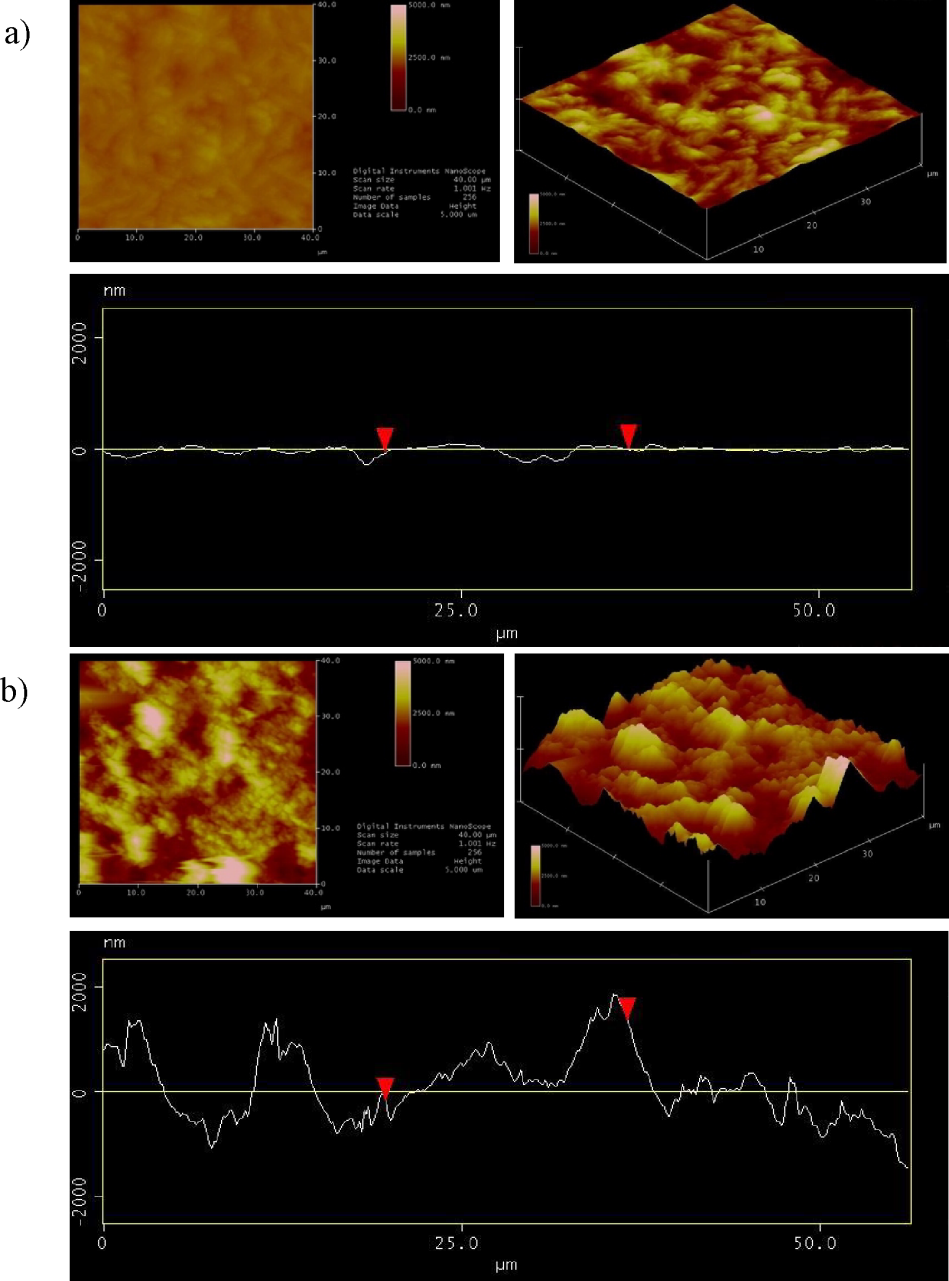
2D and 3D AFM images and cross-sectional analysis of synthesized PS thin films: (a) before and (b) after CO_2_.

AFM 2D, 3D, and cross-sectional analysis images of the synthesized PS thin films before and after the use of CO_2_ are given in Figure 5. The AFM images show that the surface roughness increased after scCO_2_ annealing. It is known that hydrophobicity increases with increasing roughness.^68^ One of the parameters controlling hydrophobicity in this system is the thin film surface roughness in addition to chain entropy.

The specificity of the membranes synthesized in this study is based on their selectivity in gas-moisture permeability and the CO_2_ annealing process used to achieve this selectivity. Film thickness and PEG ratio affect the PS/PEG distribution and the porosity and stability of the resulting structure. Considering the molecular sizes of the analyte gases and water molecules, it was predicted that the membrane structure with the minor porosity and homogeneous distribution of the related porosity would be ideal. Accordingly, oxygen and moisture permeability measurements are given in Table 1 for undoped PS, PS/PEG (10:1), and CO_2_ annealed undoped PS and PS/PEG (10:1) samples. The adverse effects of ambient oxygen on the sensor performance of gas sensors operating at room temperature are known.^1^ The synthesized membranes were, therefore, supposed to be selectively permeable, allowing oxygen to get through as much as possible while keeping moisture out.^58^ The oxygen permeability test was applied to various PS-based membrane structures synthesized as PS/PEG and obtained by removal of PEG from the PS chains in pure water. The permeability of the 500 nm film is significantly impacted by microporosities. Specifically, PEG removal increases the oxygen permeability before CO_2_ by 4.5 times compared to PS homopolymer. Following scCO_2_ annealing, oxygen permeability dropped by 30–40% as pore diameters decreased.

**Table 1:** Oxygen and Moisture Permeability Values of the Synthesized Membranes

**membrane**	**O**_**2**_**permeability (mL cm m**^**–2**^**day**^**–1**^**)**	**moisture permeability (g cm m**^**–2**^**day**^**–1**^**)**
PS-pre-CO_2_	10.1 ± 0.02	0.1 ± 0.01
PS-post CO_2_	8.4 ± 0.01	0.03 ± 0.03
PS/PEG-pre-CO_2_	45.4 ± 0.03	0.4 ± 0.02
PS/PEG-post CO_2_	34.8 ± 0.02	0.05 ± 0.01

The increased porosity with PEG removal also rapidly increased the moisture permeability. Especially in the samples with microporosity, the pre-CO_2_ values were maximized as 0.4 g cm m^–2^ day^–1^ (Table 1). The reason for this is the open porosities up to ∼0.3–0.4 μm in diameter, which can also be seen in optical SEM images. The porosities obtained with PEG removal increase oxygen gas and moisture permeability by about four times. However, after CO_2_, moisture permeability levels in 500 nm thick films decreased by 85–90%, while oxygen gas permeability decreased by only 25%. Therefore, the membrane structure after CO_2_ is more permeable to oxygen, while it behaves relatively much more restrictively in terms of moisture permeability.

**Figure 6:**
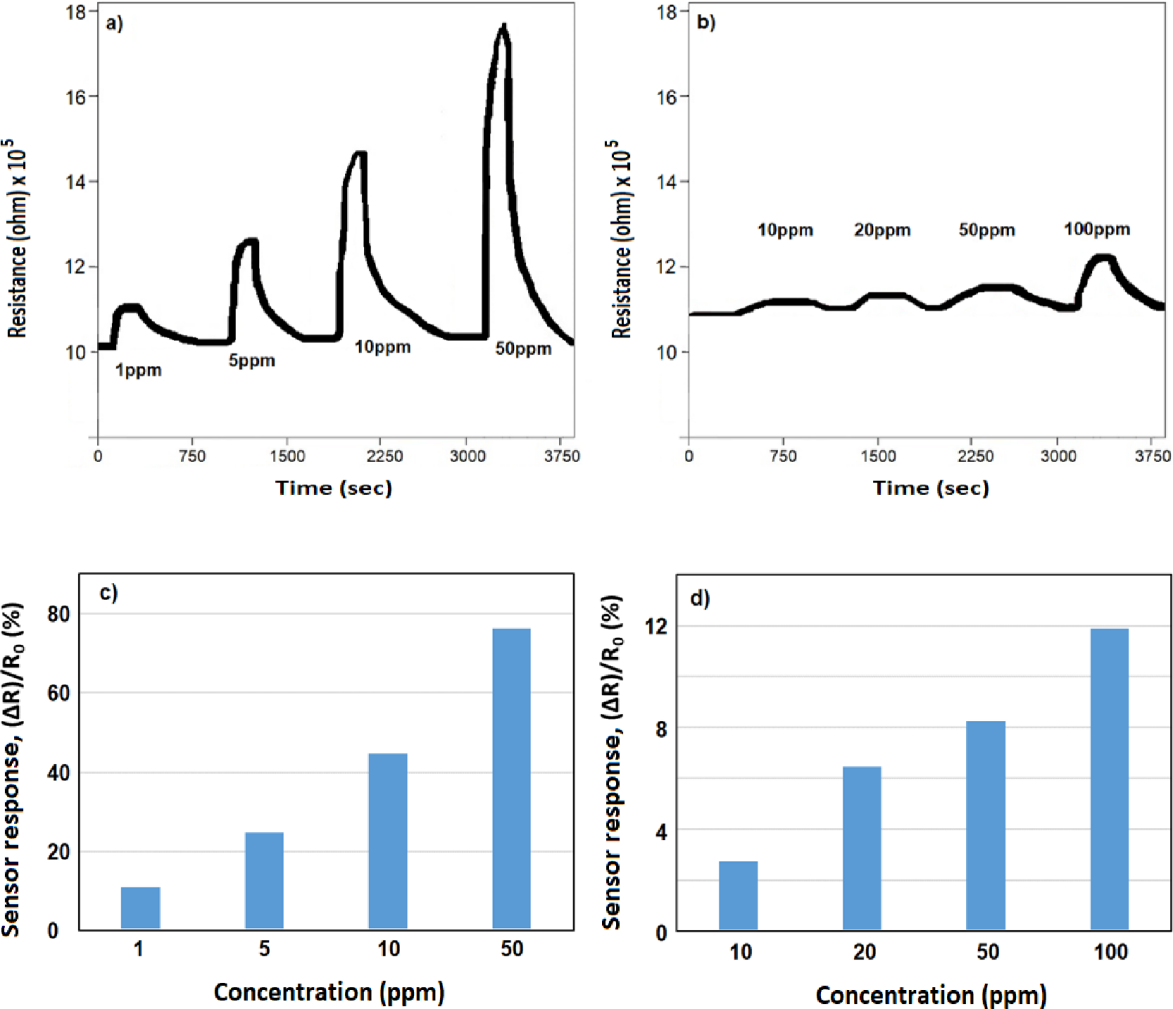
Gas sensor resistance of ZnO nanofiber structure (a) with membrane and (b) without membrane under NO_2_ target gas. Gas sensor response of ZnO nanofiber structure (c) with membrane and (d) without membrane under NO_2_ target gas.

As shown in Figure 6, the samples were tested in the gas sensor tester with and without a membrane. The resistance values obtained from the device with the membrane (Figure 6a) applied at room temperature under similar conditions were found to be ∼10.15 × 10^5^, ∼11.2 × 10^5^, ∼12.6 × 10^5^, ∼14.6 × 10^5^, and ∼17.9 × 10^5^ ohms for 0, 1, 5, 10, and 50 ppm of NO_2_ concentration, respectively. The sensor’s resistance dramatically rose within 2 min of NO_2_ entering the test chamber. Sensor resistance increased in proportion to an increase in the NO_2_ concentration. For the same sample, nonmembrane measurements revealed up to 80% reductions in sensor responsiveness for varying doses. In addition to these results, no sensor response was obtained below 10 ppm of NO_2_ in membrane-free samples (Figure 6b). The sensor responses obtained by analyzing the data obtained from the measurements according to eq 3 are given in Figure 6c,d. The concentration of NO_2_ gas has been increased gradually from 1 to 50 ppm, and the gas response also increased based on the increased concentration, indicating excellent response characteristics, and the maximum NO_2_ gas response is obtained at 50 °C. Besides, according to the graph, the gas sensor responses remained poor under 50% RH in the measurements without a membrane. In contrast, the sensor sensitivity is at a level that detects 1 ppm of NO_2_ analyte gas for the sample with the membrane.

**Figure 7:**
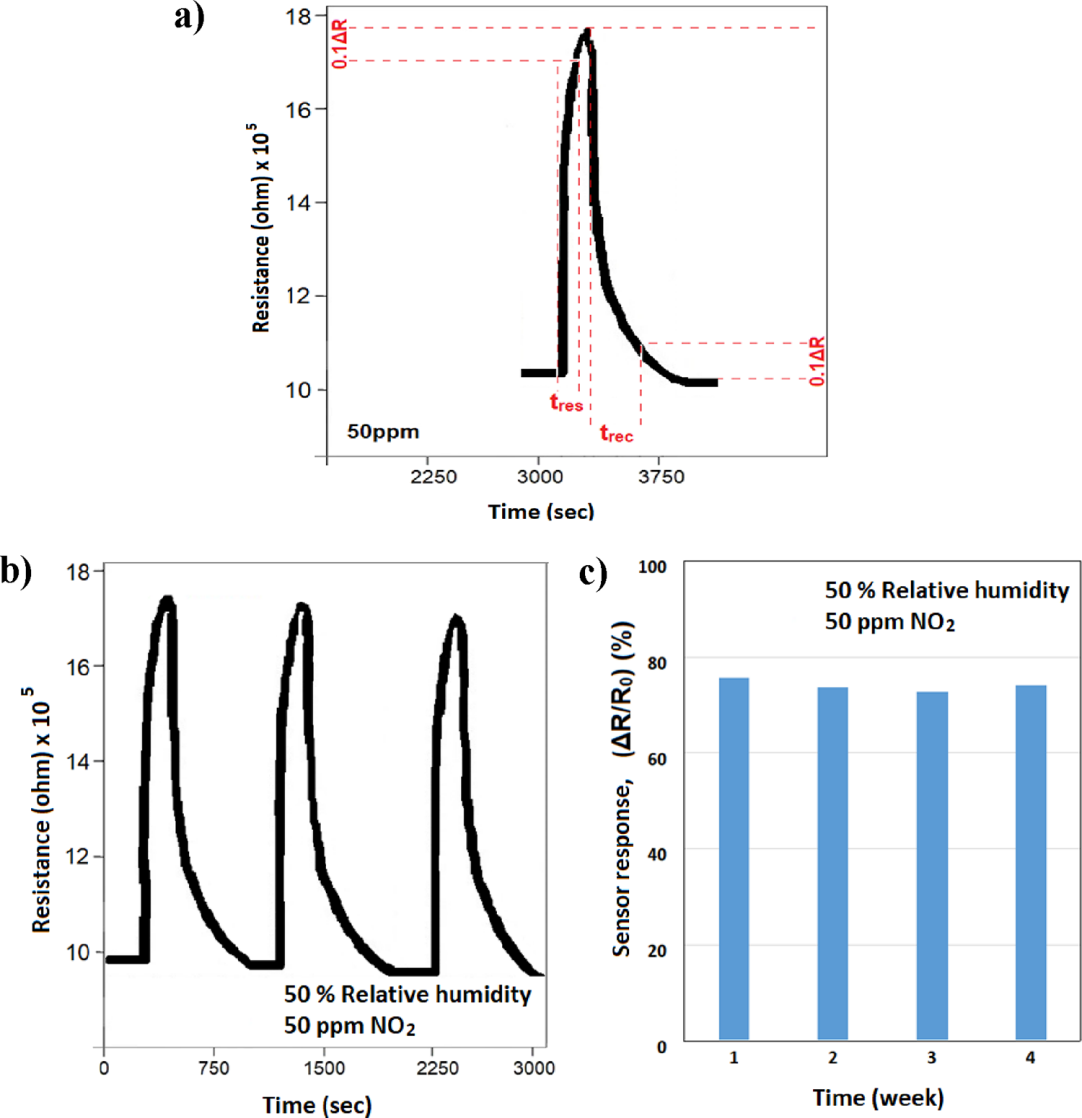
(a) Response time and recovery time, (b) repeatability, and (c) reproducibility of the response of the synthesized nanofiber gas sensor to 50 ppm of NO_2_.

The reaction time (*t*_res_) and recovery time (*t*_rec_) of the ZnO sensor for 50 ppm of NO_2_ at 50% RH for the nanofiber structures are analyzed in Figure 7a. The response time (*t*_res_) and recovery time (*t*_rec_) were 120 and 300 s, respectively.

Because of the reduced active surface area caused by surface wetness or the lower amount of O_2_ at the air interface, the resistance in the membrane-free samples remained at a certain level even when the system was swept with dry air between measurements. There was also a delay in the sensor’s regeneration. It is observed that the membrane application shortens the sensor’s self-renewal period and increases its sensitivity.

The reproducibility response of the synthesized nanofiber gas sensor with membrane at room temperature when continuously exposed to 50% RH and 50 ppm of NO_2_ gas for three consecutive cycles is shown in Figure 7b. The sensor structures repeatedly show similar sensing responses (*R*_o_ to *R*_g_ and *R*_g_ to *R*_o_) to 50 ppm of NO_2_ gas over three consecutive cycles, and the variation between measurements does not exceed 3% (Figure 7a). This result confirms the good reproducibility of the sensor measurements. In addition, measurements were repeated once a week for 1 month with the same sensor sample. The difference between the obtained sensor responses remained below 5% (Figure 7b). This difference showed an increasing trend for 3 weeks and a recovery in the sensor response in the 4th week (Figure 7c).

**Figure 8:**
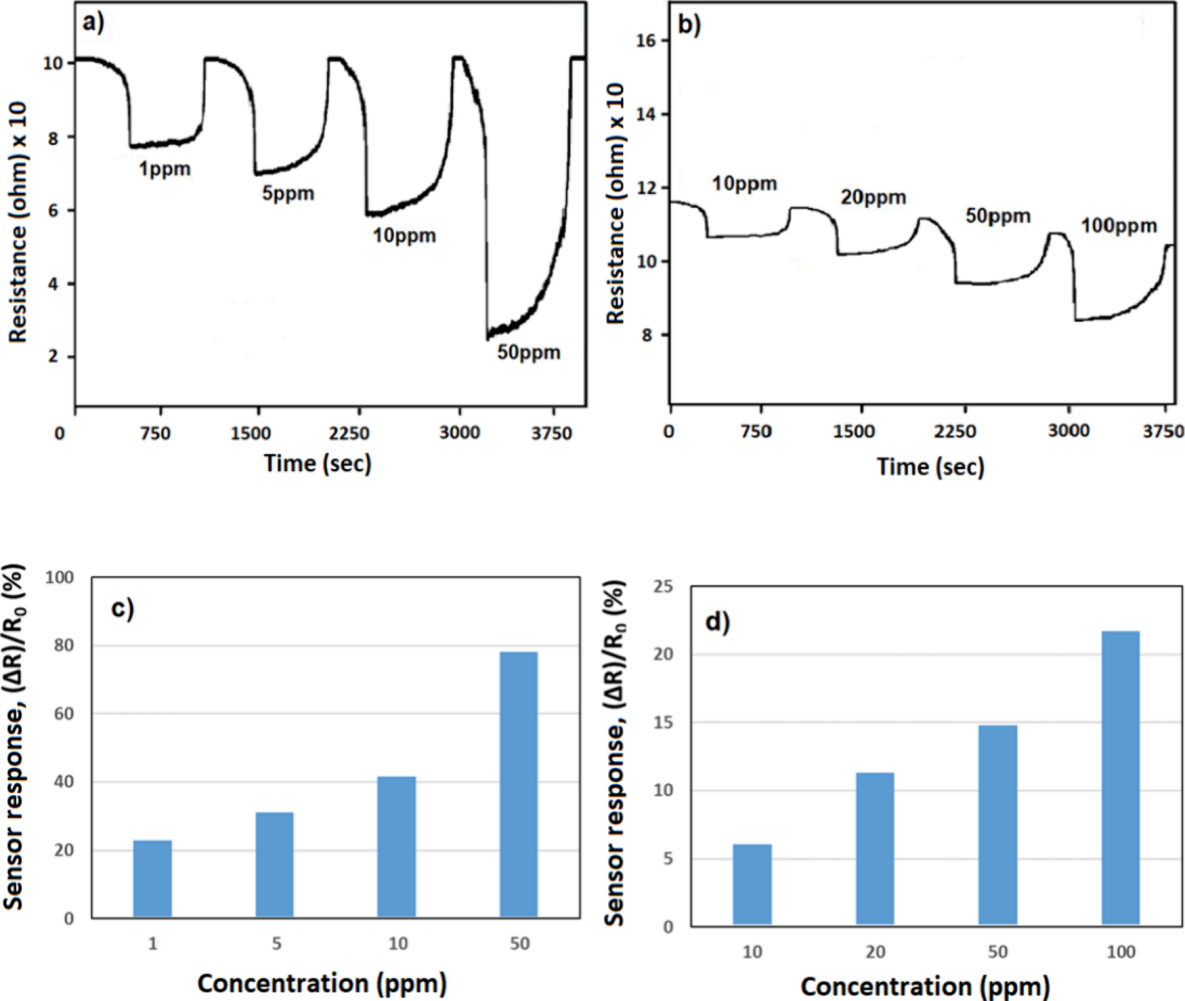
Gas sensor resistance of ZnO nanofiber structure (a) with membrane and (b) without membrane under H_2_ target gas. The gas sensor response of the ZnO nanofiber structure (c) with membrane and (d) without membrane under the H_2_ target gas.

In contrast to NO_2_, H_2_ gas was determined as the target analyte gas as a reducing gas. Figure 8a,b shows the gas sensor test results of the nanofiber structures synthesized by electrospinning and calcined at 650 °C for 2 h with membrane and without membrane. Unlike NO_2_ gas, H_2_ gas molecules, which have a reducing character compared to a ZnO sensor material, caused a decrease in sensor resistance during measurement. Based on the change in sensor resistance, it was determined that membrane application increased the sensor’s sensitivity to H_2_ analyte gas from 10 to 1 ppm. The resistance values of the membrane (Figure 8a) applied sample under 0, 1, 5, 10, and 50 ppm of H_2_ gas were found to be ∼10.1 × 10^5^, ∼7.7 × 10^5^, ∼6.8 × 10^5^, 5.9 × 10^5^, and ∼2.4 × 10^5^ ohms, respectively. Repeated measurements with the removal of the membrane show a loss of about 75% in the sensor response.

In addition, it was observed that the sensor resistance, which decreased with H_2_ gas in consecutive measurements without a membrane, did not recover completely after the gas was cut off, and a permanent loss of resistance of around 20% was observed after the experiment concluded. Comparable results to NO_2_ gas were obtained in membrane measurements under the H_2_ analyte gas. However, when the slopes of the graphs are considered, different mechanisms can be mentioned. Figure 8c,d shows the response of the ZnO sensor toward different concentrations of H_2_ gas at room temperature. The concentration of H_2_ has increased gradually from 1 to 50 ppm. A progressive increase in H_2_ gas concentration from 1 to 50 ppm was followed by a decrease in the gas response, demonstrating response characteristics. The maximum H_2_ gas response was achieved at 50 ppm. Furthermore, the graph shows that the gas sensor responses were still subpar in the measurements without a membrane at 50% RH, as mentioned in NO_2_ gas, and the lowest detectable concentration of H_2_ is 10 ppb.

**Figure 9:**
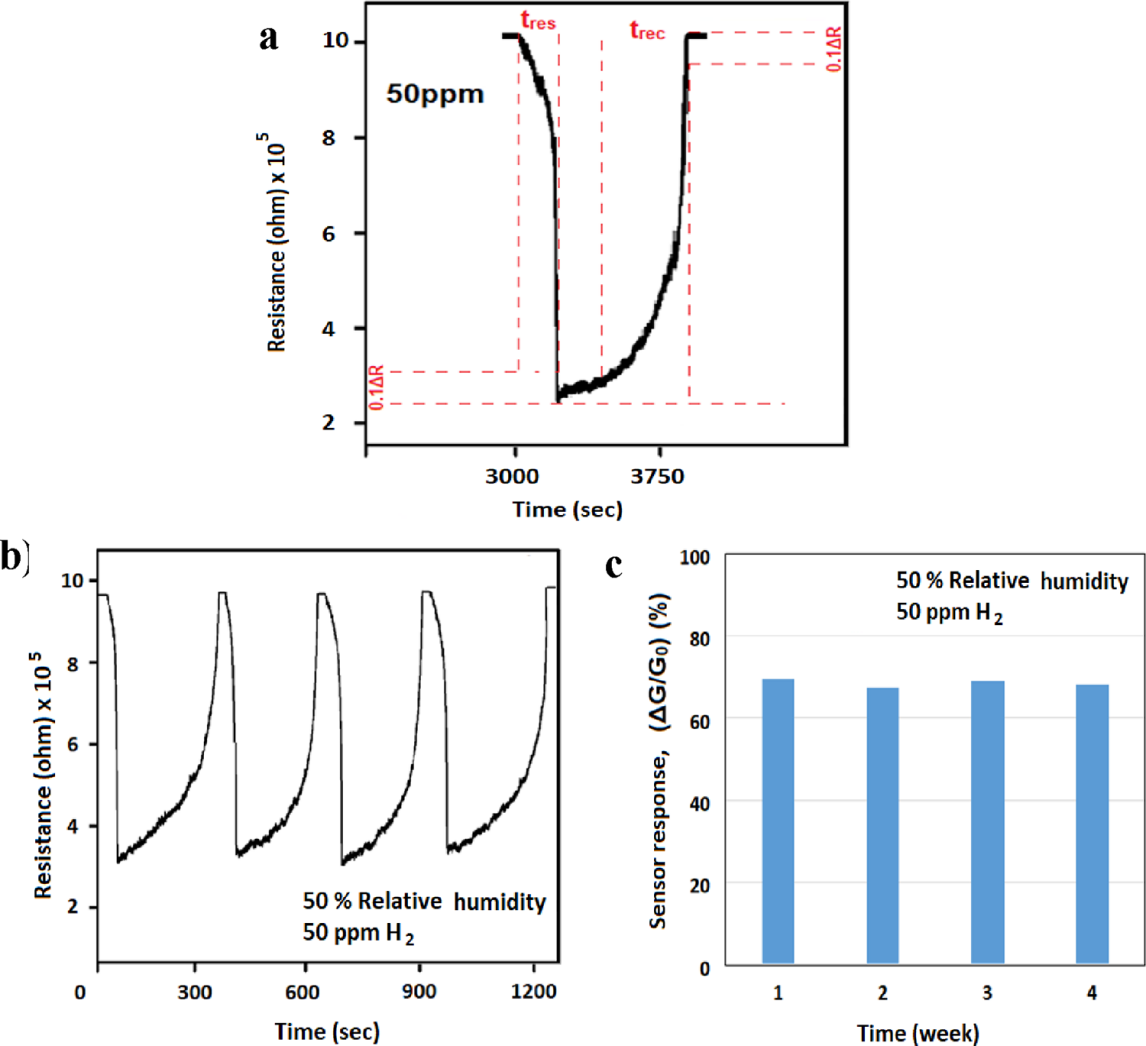
(a) Response time and recovery time, (b) repeatability, and (c) reproducibility of the response of the synthesized nanofiber gas sensor to 50 ppm of H_2_.

Based on the aforementioned results, Figure 9a displays the ZnO nanofiber sensor’s reaction time (*t*_res_) and recovery time (*t*_rec_) for 50 ppm of H_2_. There were 220 s of response time (*t*_res_) and 400 s of recovery time (*t*_rec_). Compared to NO_2_ gas, the membrane gas sensor’s reaction and recovery times for H_2_ gas are longer. After sensor resistance reached balance, lateral behavior was observed for 60 s. Under H_2_ gas, the gas flow was stopped. Samples with a membrane show a more dynamic response to the gas flow interruption because resistance increases right after intervention. Samples without membranes started to show the same reaction within a 10 min delay, as can be seen in the graphs.

The nanofiber gas sensors shown in Figure 9b,c were used to test the repeatability of the responses to 50 ppm of H_2_ gas delivered in four consecutive cycles with membrane application. For the H_2_ analyte gas, the membrane-applied nanofiber gas sensors showed excellent repeatability. According to the sensor response, weekly repeatability measurements for 1 month demonstrated consistency with a maximum variance of 4% (Figure 9c).

**Figure 10:**
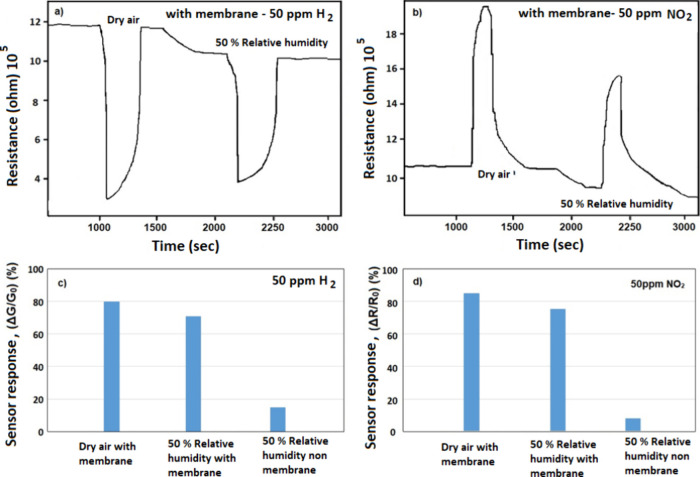
Dry air and humid air sensor measurement results applied to nanofiber-structured sensors for panels (a–c) H_2_ analyte gas and panels (b–d) for the NO_2_ analyte gas.

The test results for dry and humid air supplied to the nanofiber sensors are displayed in Figure 10. These findings indicate that the sensor response is decreased by roughly 15% when moisture is present in the membrane samples. This loss reaches up to 90% in the membrane-free samples for different systems from the literature.^69^

In Table 2, the results of this study are presented together with data from some literature studies. The data show that sensor performance can be improved with membrane application. On the other hand, sensor response dynamics with and without membranes differ, as explained above. Because the gas flow through the membrane is not linear, there is fluctuation due to moisture accumulation or the distribution of pores over the barrier surface.

**Table 2:** Summary of Room-Temperature Gas-Sensing Performances of the ZnO Nanofiber

**materials**	**membrane with or non**	**dry air or 50% RH**	**target gas**	**gas cont. ppm**	**response, %**	**sensitivity ppm**	**ref.**
ZnO	non		NO_2_	1	50		(70)
ZnO	non		H_2_	2000	4.92		(71)
ZnO	non	50% RH	NO_2_	100	11	0.0011	This work
ZnO	non	50% RH	H_2_	100	23	0.0023	This work
ZnO	with	dry air	NO_2_	50	84.3	0.0168	This work
ZnO	with	dry air	H_2_	50	79.5	0.0159	This work
ZnO	with	50% RH	NO_2_	50	76.3	0.0152	This work
ZnO	with	50% RH	H_2_	50	76.2	0.0152	This work

Compared to other methods, the structures prepared by this method can be simple and effective in controlling the performance of gas sensors operating at room temperature. With this study, it is predicted that sensor performance can be further improved by CO_2_ annealing processes applied to the polymer membrane and changes in the structure through a structure/property relationship.

### Sensing Mechanism of the ZnO Nanofiber

A depletion layer known as a space charge layer forms on the ZnO sensor’s surface when exposed to air. This increases the material’s resistance because oxygen molecules adsorb on the surface of the materials to form chemisorbed oxygen anions (O_2(ads)_^–^) by capturing electrons from the conduction band.
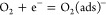
5

NO_2_ gas is instantly adsorbed on the ZnO surface when it comes into contact with the ZnO nanowire. Consequently, the resistance of the ZnO layer rises as the electron concentration on the ZnO surface falls. The following is a description of the reaction process:
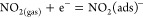
6

The above reactions decrease the electron concentration on the ZnO surface, increasing the material’s resistivity. In addition, the following reaction occurs between NO_2(ads)_ and O_(ads)_:
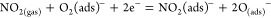
7

Hydrogen molecules will combine with oxygen ion species adsorbed on the ZnO surface to generate H_2_O molecules when the gas sensor is exposed to reduced gases, such as hydrogen. The previously caught electrons are released back into the ZnO conduction band during the chemical process, increasing the electron concentration and lowering the sensor’s resistance. The ZnO surface’s potential barrier and the void layer will get thinner simultaneously. The following is an expression for the entire reaction:
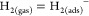
8
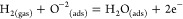
9
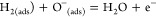
10

During the gas sensor test phases, it is observed that different sensor responses are obtained with the same membrane application. As the sensor resistance increases or decreases based on the analyte gas, it is observed that the resistance shifts from parabolic to linear or from linear to parabolic, particularly when the analyte gas is introduced into the system. It can be seen that this trend varies under different gases with the same device and membrane. This is believed to be caused by differences in the target gas’s diffusion from the membrane to the system and changes in the carrier gas and RH. For instance, while H2 has an apolar structure in contrast to the PS membrane, which is known to be apolar but contains partial polarity, water and NO_2_ molecules exhibit polar behaviors. Apart from these structural variations, it is known that a complex dynamic structure is created depending on the gases’ molecular sizes and the membrane’s porosity and distribution. As a result, it is believed that this study can be continued with additional research on the relevant gas diffusion mechanics and related sensor test equipment design.

## 4 Conclusions

Within the scope of this study, ZnO structures and synthesized nanofiber structures were electrospun and a selectively permeable polymer film was applied to them. PS/PEG membranes were produced based on water repellency and/or physical nano/microporosity for the free polymer membrane film. Optimum membranes for optimum analyte gas and minimum moisture permeability were obtained by CO_2_ annealing the synthesized membranes. The nanometer-sized and homogeneously distributed pore structures of the films after CO_2_ are a first in the literature.

With the designed noncontact gas sensor test setup, membrane gas sensor tests were performed against both NO_2_ and H_2_ gases. The response of this sensor toward NO_2_ gas (50 ppm) reached 76.3, with response and recovery times of 120 and 300 s, respectively. The sensor’s tremendous potential for real-world applications is demonstrated by its good repeatability. With response and recovery times of 220 and 240 s, respectively, this sensor’s response to H_2_ gas (50 ppm) was 76.2. Due to both the sizable length-to-diameter ratio and the high surface-to-volume ratio from their 1D hollow nanostructure and membrane applications, the ZnO nanofibers would exhibit good sensing characteristics.

As a result, this study is thought to provide a new approach to the literature within the scope of reducing the effects of RH on the sensor, which is one of the biggest problems in sensors operating at room temperature.
